# Phase I and pharmacologic study of CT-2584 HMS, a modulator of phosphatidic acid, in adult patients with solid tumours

**DOI:** 10.1054/bjoc.2000.1503

**Published:** 2000-12

**Authors:** S L Cheeseman, M Brannan, A McGown, P Khan, C Gardner, L Gumbrell, D Dickens, M Ranson

**Affiliations:** CRC Department of Medical Oncology, Christie Hospital NHS Trust, Manchester; Clinical Pharmacology, Cell Therapeutics Inc, Seattle, WA; CRC Section of Drug Development and Imaging, Paterson Institute for Cancer Research, Wilmslow Road, Manchester, M20 4BX; Cancer Research Campaign, 10 Cambridge Terrace, London, NW1 4JL, UK

**Keywords:** CT-2584 HMS, phosphatidic acid, Phase I, pharmacokinetic

## Abstract

CT-2584 HMS, 1-(11-dodecylamino-10-hydroxyundecyl)-3,7-dimethylxanthine-hydrogen methanesulphonate, is a modulator of intracellular phosphatidic acid. We treated 30 patients as part of a Phase I and pharmacokinetic study to determine the maximum-tolerated dose of CT-2584 HMS, toxicity profiles, pharmacokinetic profile and antitumour effects at escalating dose levels. CT-2584 HMS was given as a continuous infusion for 6 hours for 5 consecutive days every 3 weeks. Plasma samples for pharmacokinetic studies were analysed using a validated high-performance liquid chromatographic assay. Mean C _max_ and AUC values for each dose group were similar on days 1 and 5 and increases in plasma concentration (C _max_ and AUC) appeared proportional to the dose. CT-2584 HMS had a mean elimination half-life of 7.3 hours. Values of V _d_ and clearance were independent of dose and duration of treatment. Dose escalation was halted at 585 mg/m^2^ because of malaise and lethargy, which was sometimes accompanied by nausea and headache. 26 patients were evaluable for response, one patient with pleural mesothelioma achieved a partial response to treatment confirmed by CT scanning. A dose level of 520 mg/m^2^ daily × 5 days would be suitable for Phase II testing. Alternative schedules of CT-2584 HMS to overcome the limiting toxicity of malaise would be worthy of examination. © 2000 Cancer Research Campaign http://www.bjcancer.com


*British Journal of Cancer* (2000) **83**(12), 1599–1606
© 2000 Cancer Research Campaign
doi: 10.1054/ bjoc.2000.1503, available online at http://www.idealibrary.com on IDEAL®

### Phase I and pharmacologic study of CT-2584 HMS, a modulator of phosphatidic acid, in adult patients with solid tumours

**SL Cheeseman[1], M Brannan[2], A McGown[3], P Khan[3], C Gardner[4], L Gumbrell[4], D Dickens[1] and M Ranson[1]**

[1]CRC Department of Medical Oncology, Christie Hospital NHS Trust, Manchester; [2]Director, Clinical Pharmacology, Cell Therapeutics Inc, Seattle, WA; [3]CRC Section of Drug Development and Imaging, Paterson Institute for Cancer Research, Wilmslow Road, Manchester, M20 4BX; [4]Cancer Research Campaign, 10 Cambridge Terrace, London NW1 4JL, UK

**Summary** CT-2584 HMS, 1-(11-dodecylamino-10-hydroxyundecyl)-3,7-dimethylxanthine-hydrogen methanesulphonate, is a modulator of intracellular phosphatidic acid. We treated 30 patients as part of a Phase I and pharmacokinetic study to determine the maximum-tolerated dose of CT-2584 HMS, toxicity profiles, pharmacokinetic profile and antitumour effects at escalating dose levels. CT-2584 HMS was given as a continuous infusion for 6 hours for 5 consecutive days every 3 weeks. Plasma samples for pharmacokinetic studies were analysed using a validated high-performance liquid chromatographic assay. Mean $C_{max}$ and AUC values for each dose group were similar on days 1 and 5 and increases in plasma concentration ($C_{max}$ and AUC) appeared proportional to the dose. CT-2584 HMS had a mean elimination half-life of 7.3 hours. Values of $V_d$ and clearance were independent of dose and duration of treatment. Dose escalation was halted at 585 mg/m$^2$ because of malaise and lethargy, which was sometimes accompanied by nausea and headache. 26 patients were evaluable for response, one patient with pleural mesothelioma achieved a partial response to treatment confirmed by CT scanning. A dose level of 520 mg/m$^2$ daily × 5 days would be suitable for Phase II testing. Alternative schedules of CT-2584 HMS to overcome the limiting toxicity of malaise would be worthy of examination. © 2000 Cancer Research Campaign http://www.bjcancer.com

**Keywords:** CT-2584 HMS; phosphatidic acid; Phase I; pharmacokinetic

There has been rapid advance in the understanding of aberrant cell growth in tumours in recent years and this has yielded a number of potential therapeutic targets for drug development. Second messenger signal transduction has been of particular interest. One signalling lipid that is aberrantly regulated in neoplasia is phosphatidic acid (PA). Cellular levels of PA are increased following neoplastic transformation with *ras* and *fps* oncogenes (Martin et al, 1991) and elevated levels of PA are correlated with expression of the *src* oncogene (Song et al, 1991; Song and Foster, 1993) and the MDR phenotype in tumour cells. PA is involved in calcium mobilization (Siddiqui and English, 1997), transport from the endoplasmic reticulum to the Golgi complex (Bi et al, 1997) and activation of other signalling cascades (Siddiqui and Yang, 1995; English et al, 1996). PA has been linked to the activation of the neutrophil respiratory burst enzyme, NADPH oxidase (Waite et al, 1997), and is also a potent mitogen, and may exert its effects through the action of the small G-proteins (Exton, 1997). The potential therapeutic index for PA modulators is high, as normal cells under homeostatic conditions have very low levels of PA, malignant transformed cells express high levels of PA.

CT-2584 HMS, 1-(11-dodecylamino-10-hydroxyundecyl)-3,7-dimethylxanthine-hydrogen methanesulphonate, is a cytotoxic agent which modulates intracellular metabolism of phosphatidic acid in tumour cells (Singer et al, 1994; Eiseman et al, 1995). CT-2584 HMS demonstrated activity against a wide range of human tumour cells in vitro; lung, colon, melanoma, renal, ovarian, breast, brain, prostate and leukaemia. National Cancer Institute (NCI) toxicity testing using the NCI's preclinical antitumour drug discovery screen on cell lines derived from 9 human tumours gave a mean $LC_{50}$ of 6.14 ± 1.70 μM. The mean concentration of CT-2584 HMS that produced growth inhibition of 50% cells ($GI_{50}$) was 1.68 ± 0.76 μM and a mean concentration of CT-2584 HMS that produced total growth inhibition (TGI) was 3.20 ± 0.64 μM. CT-2584 HMS was cytotoxic to cell lines resistant to commonly used cytotoxic drugs; cisplatin, adriamycin and paclitaxol. With overnight exposure to CT-2584 HMS the mean $LC_{50}$ value of human tumour cell lines resistant to conventional chemotherapeutic agents was 4.41 ± 3.78 μM (median 3.29 μM, range 1.33–11.57 μM) (data on file, Cell Therapeutics Inc, Seattle, USA). In contrast, CT-2584 HMS is relatively non-toxic to haemopoietic cells with a mean $LC_{50}$ value of 16.26 μM (range 7.73–39.38 μM).

CT-2584 HMS shows antitumour activity in mice with established subcutaneous B16 melanoma and established Lewis lung carcinoma pulmonary metastases. In direct comparisons to etoposide at its MTD, CT-2584 HMS was as, or more, effective at suppressing the growth of B16 melanoma and NCI H460 human lung cancer xenographs.

The effect of CT-2584 HMS on the growth of a variety of tumour cells taken directly from patients was tested in a human tumour soft agar cloning assay. 24-hour exposure to CT-2584

*Received 16 December 1999*
*Revised 6 July 2000*
*Accepted 9 August 2000*

*Correspondence to:* SL Cheeseman

HMS at concentrations of 5 and 20 μM inhibited growth of 12 and 65% of tumour cells, respectively.

Preclinical studies were carried out in rats, beagles and non-human primates. In Sprague Dawley rats, red urine was observed at doses of 150 mg/m$^2$ and above and was associated with a fall in haemoglobin and a rise in bilirubin, consistent with intravascular haemolysis. The $LC_{50}$ in rats of CT-2584 HMS delivered as a single 6-hour infusion through the jugular vein was between 650 and 1000 mg/m$^2$. Similar toxicities were seen in Beagles treated at 200, 400 and 800 mg/m$^2$ day$^{-1}$ × 5 days. In addition, the dogs exhibited a robust hypersensitivity reaction to Cremophor®EL and were therefore pre-medicated with Benadryl® 1 hour prior to dosing with CT-2584 HMS. Non-human primates treated at 1500 mg/m$^2$ over 6 hours a day for 5 consecutive days became hypotensive, lethargic and developed haematuria and emesis. The animals recovered several hours after the treatment was discontinued. Doses of 200 mg/m$^2$ day$^{-1}$ did not result in biologically significant clinical observations.

CT-2584 HMS was selected for development on the basis of its broad antitumour activity and its favourable therapeutic ratio in tumour cells compared to normal cells. Relatively prolonged exposure to CT-2584 HMS was associated with increased in vitro cytotoxicity. The primary objectives of the study were to determine the maximum tolerated dose (MTD) and safety profile of CT-2584 HMS and to determine the pharmacokinetic profile of CT-2584 HMS at escalating dose levels. The secondary objective was to evaluate the antitumour effects of CT-2584 HMS. Preclinical toxicity studies showed an $LD_{10}$ in rats of 650 mg/m$^2$ and a ‘no effect level’ in monkeys (*Macaca fascicularis*) of 200 mg/m$^2$.

The target therapeutic dose of CT-2584 HMS is one that would generate a blood level of 3–5 μM over a 4 to 6 hour infusion period. This level and duration are based on in vitro cell cytotoxicity data. Stable plasma levels of 0.50 μM were achieved with a 24-hour infusion of 490 mg/m$^2$ and plasma levels of 2.27 μM were achieved with 350 mg/m$^2$ over 3 hours for 3 consecutive days in non-human primates.

A Phase I trial using CT-2584 HMS administered intravenously via a central venous catheter for 6 hours daily for 5 consecutive days repeated every 3 weeks was designed to study the pharmacologic profile and determine the dose-limiting toxicity in adult patients with solid malignancies. Based on the $LD_{10}$ in rats of 650 mg/m$^2$ and the ‘no effect level’ in non-human primates of 200 mg/m$^2$ an initial starting dose of 65 mg/m$^2$ was proposed. This starting dose was chosen to allow a reasonable margin of safety with the goal of exceeding the proposed therapeutic blood level within the dose escalation scheme proposed.

### PATIENTS AND METHODS

### Patient selection

Patients over 18 years of age with a histologically proven cancer refractory to conventional therapy or for which no standard therapy existed were eligible for the trial. Other eligibility criteria were: Karnofsky score of > 70; expected survival ≥ 12 weeks; no previous anticancer therapy for 4 weeks (6 weeks for nitrosourea or mitomycin C); creatinine ≤ 130 μmol l$^{-1}$: total bilirubin ≤ 20 μmol l$^{-1}$; serum AST and ALT <2 × upper limit of normal range; WBC ≥ 3.0 × 10$^9$ l$^{-1}$; platelet ≥ 100 × 10$^9$ l$^{-1}$, haemoglobin ≥10.0 g dl$^{-1}$ and absolute neutrophil count of >1.0 × 10$^9$ l$^{-1}$. Patients with major thoracic or abdominal surgery within 4 weeks prior to study drug, cerebral metastases or a history of seizures, hepatitis B or C, or patients receiving anticoagulants were excluded from the study. All women of childbearing age were required to have a negative pregnancy test and to use adequate contraception. The structural similarity between CT-2584 HMS and methylxanthines suggested that CT-2584 HMS might have methylxanthine-like side effects such as angina, hypotension and arrhythmias. A preliminary preclinical pharmacological profile confirmed CT-2584 HMS to exhibit mild anti-hypertensive and anti-arrhythmic properties. For this reason patients with significant cardiovascular disorders, or patients taking concurrent digoxin therapy were excluded from the study. Experimental drugs within 28 days prior to dosing were not permitted. Patients had recovered from all side effects of prior treatment. Informed written consent was required, and the placement of central venous access for CT-2584 HMS administration. The protocol was approved by the South Manchester Research Ethics Committee, and conducted under the auspices of the CRC Phase I/II Committee. The trial was conducted to the requirements of Good Clinical Practice Guidelines.

### Treatment and dose escalation

CT-2584 HMS was manufactured by Sanofi Winthrop, Kansas, USA and supplied by BlisTech Corporation, Fairfield, New Jersey, USA for Cell Therapeutics Inc, Seattle, Washington, USA.

CT-2584 HMS injection was provided as a sterile solution of CT-2584 HMS in water for injection at a concentration of 20 mg ml$^{-1}$. The drug was formulated in Cremophor® EL at a concentration of 10% v/v. The solution was diluted in 5% dextrose to a concentration of 2 mg ml$^{-1}$ prior to administration, the solution being stable at room temperature for 24 hours. CT-2584 HMS is a marked venous irritant (pH 3.75) and the infusion was administered as a continuous infusion through a tunnelled central venous catheter. This was placed in the subclavian vein several days prior to commencing therapy on trial.

CT-2584 HMS at a starting dose of 65 mg/m$^2$ was given for 6 hours daily for 5 days. Each daily treatment was commenced in the morning, beginning on day 1. A three-week cycle of CT-2584 HMS consisted of 5 days of CT-2584 HMS administration (Day 1–Day 5). Patients were monitored during each infusion and then weekly. No intra-patient dose escalations were permitted and the protocol did not allow dose modifications for individual patients. A minimum of three patients were treated at each dose level. If one of the three patients developed a dose limiting toxicity (DLT), three additional patients were treated at that dose level. Dose escalation was based on a modified Fibonacci Search Scheme. Dose limiting toxicity was defined as grade 4 thrombocytopenia and/or neutropenia or grade 3 non-haematologic toxicity excluding nausea, vomiting and alopecia. Dose levels of 65 mg/m$^2$, 130 mg/m$^2$, 215 mg/m$^2$, 325 mg/m$^2$, 455 mg/m$^2$ and 585 mg/m$^2$ per day were defined. Following completion of the 455 mg/m$^2$ dose level and review of the toxicology, a dose level of 520 mg/m$^2$ was added.

The maximum tolerated dose (MTD) was defined as the highest dose at which less than 2 of 6 patients developed a DLT. No patient was admitted at a higher dose level until all patients at a given level had reached day 22 of the first cycle.

For the purposes of the study, an evaluable patient was defined as one who met all the inclusion criteria and had no exclusion criteria, and who received at least one dose of CT-2584 HMS.

*© 2000 Cancer Research Campaign*

### Treatment procedures and observations

Before initiating therapy, a complete medical history was taken, documenting all previous surgery, chemotherapy, radiotherapy and immunotherapy. A physical examination was conducted, baseline signs and symptoms recorded and determination of Karnofsky score were recorded within one week prior to commencing therapy with CT-2584 HMS. All patients had appropriate baseline imaging and all measurable, evaluable and non-measurable tumour sites were documented within 4 weeks prior to dosing. A full blood count (FBC), including differential white cell count, biochemical screen, coagulation studies (prothrombin time and partial prothrombin time), hepatitis B and C serology, urinalysis, electrocardiogram (ECG) and chest X-ray were performed in all patients prior to commencing therapy. A pregnancy test was performed in women with childbearing potential. During the treatment week, patients had daily complete physical examination and documentation of Karnofsky score, FBC, biochemistry, urinalysis pre- and post-infusion and assessment of toxicity. Coagulation studies were obtained on day 1 and day 5. Toxicity was defined using the National Cancer Institute of Canada Clinical Trials Group (NCIC-CTG) Expanded Common Toxicity Criteria (CTC – version 1.0). An ECG was obtained before each CT-2584 HMS administration. Tumour evaluation was performed after every two cycles of CT-2584 HMS. Tumour marker levels were assessed after every second cycle of CT-2584 HMS in patients who exhibited elevated levels during the pre-treatment period. Patients with stable disease or better could receive up to 6 cycles of study treatment.

### Sample collection and drug analysis

Blood samples for assessment of CT-2584 HMS concentrations were collected on cycle 1, days 1 and 5. Sample times were immediately prior to commencing the infusion, 1.5 and 5.5 h into the infusion and immediately at the completion of infusion, 15, 30, 60 min and 2, 4, 6, 12 and 18 h after the completion of the 6-hour infusion. A 72-h sample was also taken after the completion of therapy on day 5. Samples (~4.5 ml) were taken into tubes containing sodium citrate and placed on ice. Each sample was then centrifuged at 3000 rpm for 20 minutes and the supernatant removed and frozen at –20˚C prior to analysis. CT-2584 HMS content was determined by the Bioanalytical Chemistry Department of Covance Laboratories Inc, Wisconsin using an HPLC method with ultra-violet (UV) detection. A validation system was developed 'in house' and duplicate samples analysed (Khan et al, 1999). CT-2584 HMS was extracted into potassium phosphate extraction buffer and methyl-t-butyl ether from human plasma. The organic layer was transferred to a clean test tube and brought to dryness before reconstitution with acetonitrile:sodium chloroacetate buffer and analysed by HPLC with UV detection. The peak height ratio of CT-2584 HMS relative to the peak height of internal standard was determined and a weighted least squares linear regression analysis was performed. The calibration curve was then used to calculate the concentration of CT-2584 HMS in the patient and QC samples. The lower limit of quantitation for CT-2584 HMS was 20 ng $ml^{-1}$. Samples from 4 patients were thawed during shipment, and in 2 patients, high internal standard responses were obtained which resulted in analysis failure. Results from the final 2 patients await analysis.

In 5 patients, multiphasic curves were obtained with CT-2584 HMS and this precluded non-compartmental analysis. A complete pharmacokinetic profile ($C_{max}$, AUC, $T_{1/2}$, $V_d$ and CL) was determined for each evaluable patient using a non-compartmental model and *WinNonlin Professional 2.1* software (Pharsight Corporation, Cary, NC, USA).

### RESULTS

A total of 30 patients were enrolled into the study, 13 males and 17 females. 28 patients had a Karnofsky score of 80 to 90, 1 patient each had scores of 100 and 70. Patients had a wide range of malignancies, and as expected, most patients had received several prior modalities of treatment. 7 patients had not received prior chemotherapy or radiotherapy and all but one of these had advanced inoperable mesothelioma. Patient characteristics are summarized in Table 1.

The safety of CT-2584 HMS was examined at 7 dose levels from 65 mg/m$^2$ × 5 days to 585 mg/m$^2$ × 5 days. Preclinical toxicity studies suggested that CT-2584 HMS could cause anorexia, decreased activity, haemolysis (presenting with reddening of the urine) and hypersensitivity reactions and at high doses, vomiting and convulsions. Catheter-related complications were common. In the Phase I trial, toxicity was uncommon below 325 mg/m$^2$ × 5 days. At 455 mg/m$^2$ × 5 days, 1 patient experienced grade 3 nausea and grade 2 vomiting. Grade 2 anorexia was noted in 1 patient. Cardiovascular side effects were also seen at this dose level with 2 patients developing hypotension and one patient cardiac ischaemia related to the study drug, drug-related malaise was noted in 5 of 6 patients and this was grade 2 or less. No further drug-related cardiac toxicity was noted at higher dose levels. At a dose of 520 mg/m$^2$ × 5 days, malaise became more marked, with 4 out of 6 patients developing grade 2 or 3 toxicity. At 585 mg/m$^2$ × 5 days, grade 3 malaise and grade 4 vomiting was seen in 1 patient. At this dose, two of three patients discontinued treatment because of drug-related toxicity and continuation of the study was deemed inappropriate. Drug-related toxicities are described in more detail below and summarized in Table 2.

### Gastrointestinal toxicity

20 patients (66%) experienced nausea and 16 (53%) experienced vomiting. Routine prophylactic antiemetics were not given with the first cycle but when nausea was encountered this was easily controlled using standard antiemetics (metoclopramide or ondansetron). 14 patients suffered grade 1 or 2 vomiting, one patient, treated at 585 mg/m$^2$, developed grade 4 vomiting accompanied by grade 3 malaise, facial flushing and backache after receiving 1100 mg of day 1, cycle 2 dose of CT-2584 HMS (585 mg/m$^2$). The study drug was discontinued and the symptoms resolved over the following 3 days.

### Renal and bladder toxicity

Preclinical animal studies had shown that CT-2584 HMS therapy could cause red cell haemolysis. Urine was tested for blood, bilirubin and protein pre- and post-infusion daily during the drug infusion days. Transient proteinuria was detected in 14 patients (47%) and haematuria in 10 (33%) patients. Except in two instances, haematuria was detected only on urinalysis. Macroscopic haematuria was observed in two patients. One patient with an ovarian teratoma treated at a dose level of 520 mg/m$^2$ developed

© 2000 Cancer Research Campaign 

**Table 1** Patient details

| Pt. no. | Age/sex | Disease | No. cycles completed | Dose mg/m² | Response | Reason stopped |
|---|---|---|---|---|---|---|
| 1 | 33/M | Malignant melanoma | 2 | 65 | PD | HSR |
| 2 | 60/M | Malignant pleural mesothelioma | 6 | 65 | PR | Study completed |
| 3 | 34/F | Metastatic malignant paraganglioma | 2 | 65 | PD | Disease progression |
| 4 | 78/F | Malignant melanoma | 1 | 130 | PD | Disease progression |
| 5 | 46/F | Ovarian adenocarcinoma | 1 | 130 | PD | Disease progression |
| 6 | 46/M | Non small cell lung carcinoma (adeno) | 2 | 130 | PD | Disease progression |
| 7 | 61/M | Malignant pleural mesothelioma | 2 | 130 | PD | Disease progression |
| 8 | 64/F | Malignant pleural mesothelioma | 4 | 130 | SD | Disease progression |
| 9 | 41/M | Peritoneal mesothelioma | 4 | 130 | NE | Patient request, subclavian line malfunction |
| 10 | 45/F | Ovarian adenocarcinoma | 2 | 215 | PD | Disease progression |
| 11 | 64/F | Ovarian adenocarcinoma | 2 | 215 | PD | Disease progression |
| 12 | 66/F | Unknown primary site carcinoma | 2 | 215 | PD | Disease progression |
| 13 | 46/F | Breast carcinoma | 2 | 325 | PD | Disease progression |
| 14 | 67/F | Malignant pleural mesothelioma | 3 | 325 | SD | Sepsis and atrial fibrillation |
| 15 | 62/M | Malignant pleural mesothelioma | 2 | 325 | PD | Disease progression |
| 16 | 58/M | Malignant pleural mesothelioma | 1 | 455 | NE | Chest pain, hypotension |
| 17 | 49/M | Malignant pleural mesothelioma | 1 | 455 | PD | Patient death |
| 18 | 49/F | Ovarian adenocarcinoma | 4 | 455 | SD | Chest pain, ECG changes |
| 19 | 45/F | Ovarian cystadenocarcinoma | 1 | 455 | PD | Uncontrolled line sepsis |
| 20 | 30/M | Osteosarcoma | 2 | 455 | PD | Disease progression |
| 21 | 47/F | Inflammatory breast carcinoma | 1 | 455 | PD | Disease progression |
| 22 | 50/M | Malignant pleural mesothelioma | 1 | 520 | NE | Drug toxicity (malaise) |
| 23 | 45/F | Colonic adenocarcinoma | 1 | 520 | NE | Subclavian vein thrombosis |
| 24 | 54/F | Ovarian carcinoma | 2 | 520 | PD | Disease progression |
| 25 | 28/F | Malignant ovarian teratoma | 6 | 520 | SD | Study completed |
| 26 | 45/M | Hypernephroma | 2 | 520 | SD | Patient request |
| 27 | 71/M | Pulmonary blastoma | 4 | 520 | SD | Drug toxicity (malaise + nausea) |
| 28 | 47/M | Gastric adenocarcinoma | 3 | 585 | SD | Drug toxicity (malaise) |
| 29 | 60/F | Hypernephroma | 2 | 585 | PD | Progressive disease |
| 30 | 53/F | Soft tissue sarcoma | 2 | 585 | PD | Drug toxicity (malaise, flushing, headache) |

NE = Not evaluable; SD = Stable disease; PR = Partial response; PD = Progressive disease.

transient macroscopic haematuria which was thought to be due to CT-2584 HMS administration.

A further patient with ovarian carcinoma and bladder infiltration developed transient grade 4 haematuria and grade 2 rise in serum creatinine which was thought to be due to tumour. There were no treatment modifications or discontinuations related to these events.

### Hepatic toxicity

One patient with hepatic serosal involvement from ovarian carcinoma, had mild elevation in alkaline phosphatase (200 IU $l^{-1}$, normal range 25–100 IU $l^{-1}$) which rose to 416 by day 8 of cycle 1, and 819 by day 15 before returning to 254 by day 30. Early tumour progression was evident at day 22. A possible relationship to study drug was made.

In two patients, a transient rise in bilirubin was seen. In one patient, treated at 65 mg/m$^2$, on days 2–4 the maximum total bilirubin level was 28 µmol $l^{-1}$. This patient had also received a blood transfusion following day 1 CT-2584 HMS administration.

A further patient was noted to have a raised bilirubin of 23 µmol $l^{-1}$ (normal range 1–20 µmol $l^{-1}$) on day 5 of treatment at 455 mg/m$^2$. No clinically significant haemolytic effects or hepatic adverse events were observed in this trial.

### Haematological toxicity

CT-2584 HMS did not cause significant myelosuppression. Mild (grade 1 and 2) anaemia was common, and is likely to be due to several contributory factors; advanced cancer, compromised bone marrow function from previous therapy and frequency of blood sampling during the study. Evidence of direct haemolytic action of CT-2548 HMS has been demonstrated preclinically and minor haematuria/haemoglobinuria were observed during urinary monitoring. A small direct contribution to anaemia from CT-2584 HMS cannot be excluded.

No CT-2584 HMS related changes in white cell or platelet count were noted during this study. No significant alterations in tests of coagulation (PT/PPT) were noted.

### Cardiac toxicity

Three patients treated with CT-2584 HMS developed cardiac side effects. One patient had incomplete left bundle branch block (LBBB) on pre-treatment ECG. At completion of day 3 infusion of cycle 1 at 455 mg/m$^2$, he developed chest pain associated with sweating and grade 3 hypotension. A repeat ECG revealed complete LBBB. The patient received intravenous fluids and analgesia and his blood pressure returned to baseline levels. His ECG had returned to baseline by the following morning. Cardiac enzymes were checked and were normal. CT-2584 HMS treatment was discontinued.

Three weeks after treatment at 325 mg/m$^2$, patient #14 developed an infected leg ulcer which led to septicaemia. The patient developed atrial fibrillation associated with this event which was deemed unrelated to study drug treatment.

One patient treated at 455 mg/m$^2$, developed anginal chest pain associated with sweating and vomiting towards the end of day 5,

© 2000 Cancer Research Campaign

**Table 2** CT-2584 HMS related toxicity by dose level

| Dose mg/m² | Anorexia (grade) | | | | | Nausea (grade) | | | | | Vomiting (grade) | | | | |
|---|---|---|---|---|---|---|---|---|---|---|---|---|---|---|---|
| | 0 | 1 | 2 | 3 | 4 | 0 | 1 | 2 | 3 | 4 | 0 | 1 | 2 | 3 | 4 |
| 65 | 2 | | 1 | | | 2 | 1 | | | | 2 | 1 | | | |
| 130 | 5 | 1 | | | | 4 | 1 | 1 | | | 5 | 1 | | | |
| 215 | 2 | | 1 | | | 1 | 2 | | | | 2 | 1 | | | |
| 325 | 1 | 2 | | | | 2 | 1 | | | | 2 | 1 | | | |
| 455 | 5 | | 1 | | | 2 | 3 | | 1 | | 4 | | 2 | | |
| 520 | 4 | 2 | | | | 1 | 1 | 3 | 1 | | 2 | 3 | 1 | | |
| 585 | 2 | | 1 | | | | 1 | 1 | 1 | | | | 2 | | 1 |

| Dose mg/m² | Proteinuria (grade) | | | | | Haematuria (grade) | | | | | Malaise (grade) | | | | |
|---|---|---|---|---|---|---|---|---|---|---|---|---|---|---|---|
| | 0 | 1 | 2 | 3 | 4 | 0 | 1 | 2 | 3 | 4 | 0 | 1 | 2 | 3 | 4 |
| 65 | 2 | 1 | | | | 3 | | | | | 1 | 1 | 1 | | |
| 130 | 5 | 1 | | | | 5 | 1 | | | | 3 | 3 | | | |
| 215 | 2 | 1 | | | | 2 | | | | 1 | 1 | 1 | 1 | | |
| 325 | 3 | | | | | 3 | | | | | | 1 | 1 | 1 | |
| 455 | 3 | 2 | | 1 | | 3 | 3 | | | | 1 | 1 | 4 | | |
| 520 | 2 | 3 | 1 | | | 4 | 1 | 1 | | | 2 | | 3 | 1 | |
| 585 | 1 | 1 | 1 | | | 1 | 2 | | | | | 1 | 1 | 1 | |

| Dose mg/m² | Arrhythmia (grade) | | | | | Hypotension (grade) | | | | | Cardiac ischaemia (grade) | | | | |
|---|---|---|---|---|---|---|---|---|---|---|---|---|---|---|---|
| | 0 | 1 | 2 | 3 | 4 | 0 | 1 | 2 | 3 | 4 | 0 | 1 | 2 | 3 | 4 |
| 65 | 3 | | | | | 3 | | | | | 3 | | | | |
| 130 | 6 | | | | | 6 | | | | | 6 | | | | |
| 215 | 3 | | | | | 3 | | | | | 3 | | | | |
| 325 | 3 | | | | | 3 | | | | | 3 | | | | |
| 455 | 5 | | 1 | | | 4 | | 1 | 1 | | 5 | | | 1 | |
| 520 | 6 | | | | | 5 | 1 | | | | 6 | | | | |
| 585 | 2 | 1 | | | | 3 | | | | | 3 | | | | |

cycle 4. There were no ECG changes evident during this episode but widespread T wave inversion in V2–6 and in leads I and II developed after 36 hours. The pain responded quickly to analgesia and discontinuation of the infusion. Chest X-ray and cardiac enzymes were normal. The patient had no prior cardiac history and suffered no further cardiac sequelae. These events were judged to be related to the study drug.

### Malaise

28 of the 30 patients (93%) experienced malaise, and in a majority of instances (73%) these were attributed to the study drug. Malaise typically lasted for a few days after completion of each cycle. This was reported more frequently at dose levels of 455 mg/m² and above. Two of 3 patients at 585 mg/m² and 4 of 6 patients at 520 mg/m² developed grade 2 or worse drug-related malaise. Patient 22 developed grade 3 malaise and nausea one week after completing the first cycle of CT-2584 HMS. These symptoms had improved to grade 1 at D21 review. The patient declined further trial drug treatment. Patients 24, 27 and 28 reported grade 1 malaise at baseline which progressed to grade 2 after cycle 2 in patient 24 and cycle 3 in patients 27 and 28. In patient 27 this was associated with grade 3 nausea which required intravenous fluid replacement and antiemetics. The patient discontinued treatment.

Patient 30 developed grade 3 malaise, flushing, backache and vomiting towards the completion of D1, cycle 2 infusion. The symptoms resolved over the following 24 hours and the study drug discontinued. Significant malaise was uncommon during the first week of study drug treatment. In 4 patients malaise appeared to worsen with repeated dosing of CT-2584 HMS, suggesting a cumulative effect.

### Hypersensitivity reactions

CT-2584 HMS is formulated in Cremophor®EL, other agents formulated in this diluent can cause occasional hypersensitivity reactions (HSR). Routine hypersensitivity prophylaxis was not employed. 3 patients experienced HSR. One patient developed widespread urticaria, moderate bronchospasm, lip oedema, chest and back pain shortly after commencing day 1, cycle 2 infusion at a dose of 65 mg/m². This responded quickly to intravenous hydrocortisone and chlorpheniramine. After 1 hour a cautious rechallenge of CT-2584 HMS following premedication with dexamethasone, chlorpheniramine and ranitidine was undertaken, but resulted in a grade 2 HSR and further treatment was abandoned. A further patient, treated at 585 mg/m², developed facial flushing and backache two minutes into commencing day 1 of cycle 2 of CT-2584 HMS. Treatment with hydrocortisone and chlorpheniramine resolved the symptoms and the infusion was recommenced without further problems. Another patient treated at a dose level of 585 mg/m² developed facial flushing, malaise, backache and vomiting 5.5 h into the day 1, cycle 2 infusion. The infusion was discontinued, the flushing and backache resolved within 1 hour. The malaise improved over the following 3 days.

© 2000 Cancer Research Campaign

### Other toxicity

Headaches and dizziness related to CT-2548 HMS occurred in 30% and 27% of patients respectively.

Catheter-related sepsis was seen in 7 patients, and one patient developed a subclavian vein thrombosis.

A possible reaction with alcohol consumption was noted. Two patients reported flushing of the face and upper torso and malaise (± vomiting and headache) when consuming alcohol within 3 days of the last infusion of CT-2584 HMS.

### Pharmacokinetics

Full pharmacokinetic data were obtained in 14 patients and day 2 pharmacokinetic data were obtained in 3 additional patients (Tables 3 and 4). $C_{max}$ and AUC increased with increasing doses except for the 215 mg/m$^2$ dose group (which had only 1 patient in the analysis). Increases in CT-2584 HMS plasma concentrations appeared proportional to the dose and were similar on day 1 (Figure 1) and day 5 (Figure 2). After administration on day 1, plasma elimination half-life ranged from 5.4 to 9.6 h (mean 7.3, CV 19%) and was dose independent. Mean clearance values ranged from 66 to 78 l h$^{-1}$ on day 1 and from 46 to 78 l h$^{-1}$ on day 5. Mean volume of distribution values ranged from 607 to 1006 l on day 1 and from 466 to 822 l on day 5, exceeding the volume of total body water in humans, thus indicating that CT2585 HMS was extensively distributed in tissues. The CT-2584 HMS concentrations associated with slow release from peripheral tissues were low relative to total 24-hour exposure, and do not indicate a need for dose adjustment during 5 days' treatment with up to 520 mg/m$^2$ CT-2584 HMS.

### Responses

One partial response was seen in a male patient with pleural mesothelioma, Butchart stage I, treated at 65 mg/m$^2$ × 5 days for 6 cycles. Pleural thickening and an associated pleural effusion were visible on baseline CT scanning. After 2 cycles of CT-2584 HMS, the effusion had decreased in volume and the pleural thickening had improved by approximately 40%. After 4 cycles of CT-2584 HMS a 65% improvement was seen which was maintained after 6

**Table 3** Pharmacokinetic results for CT-2584 HMS, cycle 1, day 1

| Dose (mg/m$^2$) | | $C_{max}$ (μg ml$^{-1}$) | AUC (tf) (μg/h ml$^{-1}$) | AUC (I) (μg h ml$^{-1}$) | $T_{1/2}$ (h) | Vd (L) | Cl (L h$^{-1}$) |
|---|---|---|---|---|---|---|---|
| 65 | N analysed | 1 | 1 | 1 | 1 | 1 | 1 |
| | Value | 0.138 | 0.822 | n/v | n/v | n/v | n/v |
| | N analysed | 6 | 6 | 6 | 6 | 6 | 6 |
| 130 | Mean (SD) | 0.671 (0.60) | 4.747 (3.70) | 5.211 (3.86) | 7.3 (1.35) | 683.5 (415.77) | 65.8 (41.87) |
| | CV% | 90 | 78 | 74 | 18 | 61 | 64 |
| 215 | N analysed | 1 | 1 | 1 | 1 | 1 | 1 |
| | Value | 0.487 | 3.884 | 4.279 | 5.7 | 606.9 | 73.4 |
| | N analysed | 2 | 2 | 2 | 2 | 2 | 2 |
| 325 | Mean (SD) | 0.792 (0.06) | 6.643 (1.63) | 7.507 (1.95) | 9.1 (0.58) | 1005.6 (362.34) | 75.7 (22.70) |
| | CV% | 8 | 24 | 26 | 6 | 36 | 30 |
| | N analysed | 4 | 4 | 4 | 4 | 4 | 4 |
| 455 | Mean (SD) | 1.333 (0.24) | 10.344 (3.00) | 11.805 (3.51) | 7.1 (0.81) | 809.6 (325.93) | 78.1 (2984) |
| | CV% | 18 | 29 | 30 | 11 | 40 | 37 |
| | N analysed | 3 | 3 | 3 | 3 | 3 | 3 |
| 520 | Mean (SD) | 1.563 (0.56) | 12.664 (4.92) | 14.015 (5.37) | 6.7 (1.87) | 803.9 (539.99) | 76.6 (35.5) |
| | CV% | 36 | 39 | 38 | 28 | 73 | 46 |

**Table 4** Pharmacokinetic results for CT-2584 HMS, cycle 1, day 5

| Dose (mg/m$^2$) | | $C_{max}$ (μg/ml) | AUC (tf) (μg h/ml) | AUC (I) (μg/h/ml) | $T_{1/2}$ (h) | Vd (L) | Cl (L/h) |
|---|---|---|---|---|---|---|---|
| 65 | N analysed | 1 | 1 | 1 | 1 | 1 | 1 |
| | Value | 0.248 | 1.722 | 1.956 | 6.9 | 652.8 | 66.0 |
| 130 | N analysed | 6 | 6 | 6 | 6 | 6 | 6 |
| | Mean (SD) | 1.170 (1.47) | 6.960 (6.08) | 7.852 (6.42) | 8.7 (4.32) | 563.5 (474.95) | 46.1 (31.87) |
| | CV% | 126 | 87 | 82 | 49 | 84 | 69 |
| 215 | N analysed | 1 | 1 | 1 | 1 | 1 | 1 |
| | Value | 0.531 | 5.466 | 6.429 | 6.6 | 465.6 | 48.8 |
| | N analysed | 2 | 2 | 2 | 2 | 2 | 2 |
| 325 | Mean (SD) | 0.781 (0.14) | 6.788 (1.41) | 7.665 (1.19) | 7.0 (0.34) | 724.5 (106.76) | 72.3 (14.14) |
| | CV% | 18 | 21 | 15 | 5 | 15 | 20 |
| | N analysed | 3 | 3 | 3 | 3 | 3 | 3 |
| 455 | Mean (SD) | 1.460 (0.23) | 13.218 (3.24) | 16.136 (3.69) | 10.2 (2.19) | 821.7 (353.36) | 54.3 (12.55) |
| | CV% | 15 | 25 | 23 | 21 | 43 | 23 |
| 520 | N analysed | 1 | 1 | 1 | 1 | 1 | 1 |
| | Mean (SD) | 1.360 | 11.469 | 13.037 | 6.8 | 769.8 | 78.5 |

 *© 2000 Cancer Research Campaign*

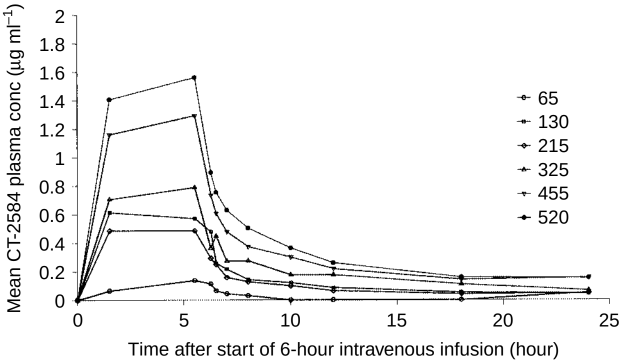

Figure 1. Mean CT-2584 HMS plasma concentration profiles by dose (mg/m²) – Day 1

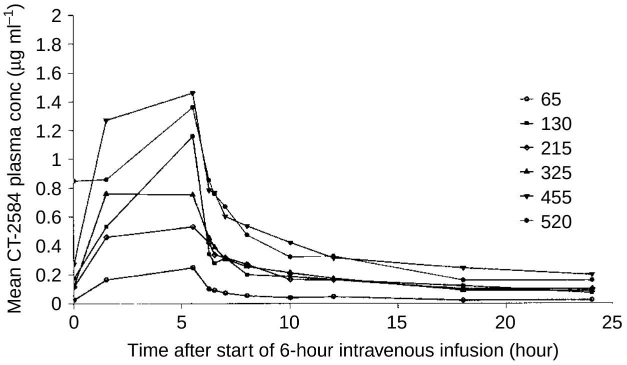

Figure 2. Mean CT-2584 HMS plasma concentration profiles by dose (mg/m²) – Day 5

cycles. The response was confirmed by CT scan and continued for 8 months. 5 further patients had stable disease for ≥12 weeks.

### DISCUSSION

Phosphatidic acid (PA) is a bioactive phospholipid which has been implicated in a wide variety of cellular events, including cellular proliferation and differentiation. CT-2584 HMS has been shown to modulate PA metabolism in tumour cells and to exhibit selective cytotoxicity to tumour cells in vitro. In vitro data suggested increased cytotoxic effect with prolonged drug exposure. Antitumour activity has been demonstrated in murine models and in human tumour xenograft models, for these reasons, CT-2584 HMS was deemed suitable for further development as a cancer therapy with a novel mechanism of action. Some PA isoforms have been found to be pro-inflammatory; PA inhibitors have been investigated as anti-inflammatory agents and also agents to promote bone marrow recovery after chemotherapy (Singer et al, 1994).

This Phase I trial was designed to examine the safety and tolerability of this drug in humans. Dose levels of 65, 130, 215, 325, 455, 520 and 585 mg/m$^2$ × 5 days were studied.

Preclinical studies suggested that CT-2584 HMS may cause anorexia, lethargy and intravascular haemolysis. No significant haematological toxicity was seen. The similarities in chemical structure between CT-2584 HMS and methylxanthines suggested that CT-2584 HMS may cause cardiovascular side effects and this was observed in 3 patients. Two patients treated at 455 mg/m$^2$ × 5 days developed cardiac side effects; one patient developed chest pain and ECG changes in keeping with cardiac ischaemia and a further patient became hypotensive and developed worsening bundle branch block during the day 3 infusion. Both patients discontinued treatment and had no further cardiovascular sequelae. A third patient developed atrial fibrillation during an episode of sepsis. Toxicities commonly seen with current cytotoxic drugs namely alopecia, bone marrow suppression and mucositis were not observed. As expected in this population of patients, grade 1 and 2 anaemia was common and multifactorial in origin. Antiemetics were not given routinely, and when treatment-related nausea or vomiting occurred it was easily controlled. Preclinical studies suggested that CT-2584 HMS could cause intravascular haemolysis. 10 patients (33%) developed transient haematuria and 2 patients had transient increases in serum bilirubin with no clinical sequelae.

CT-2584 HMS is formulated in Cremophor®EL and 3 patients experienced HSRs, in 2 patients this led to study withdrawal. Patients were not routinely given prophylactic dexamethasone prior to CT-2584 HMS infusion. The use of dexamethasone may have decreased the incidence of HSR and resultant study drug withdrawal.

Malaise appeared to be dose related and was seen in the majority of patients. In 4 patients the symptoms worsened as the treatment progressed, suggesting a cumulative effect, although this was not borne out in pharmacological studies. Treatment limiting toxicity was malaise ± nausea ± headache.

One patient died due to disease progression less than 30 days after receiving CT-2584 HMS. No drug-related deaths or irreversible toxicities were noted.

Preclinical toxicology cell line studies suggested CT-2584 HMS was lethal to 50% of cells at concentrations of 6.14 µM and inhibited growth in 50% of cells at 1.68 µm. Pharmacokinetic analysis from patients treated up to 520 mg/m$^2$ showed peak plasma concentrations were achieved towards the end of the day 1 infusion and increased between the patient cohorts with dose given. Peak plasma concentrations ranged from approximately 0.2 µg ml$^{-1}$ (65 mg/m$^2$) to 1.5 µg ml$^{-1}$ (520 mg/m$^2$). Although these figures are below $LC_{50}$ and $GI_{50}$ toxicity figures quoted from NCI toxicity studies, a patient treated at 65 mg/m$^2$ responded to this dose level. Cell toxicity studies carried out in the Drug Development Laboratories, Paterson Institute in Manchester suggest that the $GI_{50}$ is in the region of 0.2–0.8 µg ml$^{-1}$, well within the plasma levels achieved with this treatment schedule.

It was the intention of CTI to analyse patient serum samples for changes in fatty acids. Unfortunately samples were damaged in transit and were not suitable for assays on arrival. In-house PA analysis for the final 2 cohorts of patients has confirmed a fall in PA expression throughout the 5-day treatment schedule of CT-2584 HMS.

26 patients were evaluable for response. One patient, a 60-year-old male with a malignant pleural mesothelioma, achieved a partial response after 4 cycles of therapy which lasted for 8 months. Unfortunately, PA expression was not assessed during his treatment, and therefore clinical therapeutic efficiency cannot be directly compared to PA expression in this case. No other objective responses were seen. 5 patients had stable disease for at least 12 weeks during CT-2584 HMS administration.

### CONCLUSION

CT-2584 HMS was generally well tolerated at doses up 520 mg/m$^2$ when administered as a 6-hour infusion via a central venous

© 2000 Cancer Research Campaign catheter in a daily × 5 days schedule every 3 weeks. Two patients developed drug-related adverse cardiac events at 455 mg/m$^2$, but no further cardiac side effects were seen at higher dose levels. At doses of 520 mg/m$^2$ and 585 mg/m$^2$, the severity of malaise increased. The treatment limiting toxicity was malaise, sometimes accompanied by nausea and headache. It is possible that premedication with steroids may reduce these side effects. It would be of interest to study this during the proposed Phase II trials. A dose level of 500 mg/m$^2$ has been proposed for Phase II testing.

A parallel trial was undertaken in the USA, with CT-2584 HMS given as a 6-hour infusion for 3 consecutive days every 3 weeks. Results from the USA and this UK study suggest that CT-2584 HMS may be active in sarcoma and hormone-resistant prostate cancer.

### REFERENCES

Bi K, Roth MG and Ktisakis NT (1997) Phosphatidic acid formation by phospholipase D is required for transport from the endoplasmic reticulum to the Golgi complex. *Curr Biol* **7**(5): 301–307

Eiseman E, DeJongh K, Nudelman E et al (1995) Altered phosphatidic acid metabolism is implicated in selective tumour cell toxicity by the novel chemotherapeutic agent, CT-2584. *Am Soc Biochem Mol Biol* **46** (abstract)

English D, Cui Y and Siddiqui RA (1996) Messenger functions of phosphatidic acid. *Chemistry and Physics of Lipids* **80**: 117–132

Exton JH (1997) Signalling through guanine-nucleotide-binding regulatory proteins (G proteins) and phospholipases. *Eur J Biochem* **243**(1–2): 10–20

Khan P, Abbas S, Cheeseman S, Ranson M and McGown AT (1999) Development and validation of a sensitive solid-phase extraction and high-performance liquid chromatography assay for the novel antitumour agent CT-2584 in human plasma. *J Chromatogr B Biomed Sci Appl* 721: 279–284

Martin A, Gomez-Munoz A, Waggoner D et al (1991) Decreased activities of phosphatidate phosphohydrolase and phospholipase D in ras and tyrosine kinase (fps) transformed fibroblasts. *J Biol Chem* **268**: 23924–23932

Siddiqui RA and English D (1997) Phosphatidic acid elicits calcium mobilisation and actin polymerisation through a tyrosine kinase-dependant process in human neutrophils: a mechanism for induction of chemotaxis. *Biochimica and Biophysica Acta* **1349**(1): 81–95

Siddiqui RA and Yang YS (1995) Interleukin-11 induces phosphatidic acid and activated MAP kinase in mouse 3T3-L1 cells. *Cell Signal* **7**: 247–259

Singer J, Bursten S, Rice G et al (1994) Inhibitors of intracellular phosphatidic acid production: novel therapeutics with broad clinical applications. *Exp Opin Invest Drugs* **3** (6): 631–643

Song J and Foster DA (1993) V-src activates a unique phospholipase D activity that can be distinguished from the phospholipase D activity activated by phorbol esters. *Biochem J* **294**: 711–717

Song J, Pfeffer LM and Foster DA (1991) V-src increases diacylglycerol levels via type D phospholipase-mediated hydrolysis of phosphatidyl choline. *Mol Cell Biol* **11**: 4903–4908

Waite KA, Wallin R, Qualliotine-Mann D et al (1997) Phosphatidic acid-mediated phosphorylation of the NADPH oxidase component p47-phox. Evidence that phosphatidic acid may activate a novel protein kinase. *J Biol Chem* **272**: 15569–15579

 *© 2000 Cancer Research Campaign*

## References

[PDF_00831] Bi K., Roth M. G., Ktistakis N. T. (1997). Phosphatidic acid formation by phospholipase D is required for transport from the endoplasmic reticulum to the Golgi complex.. Curr Biol.

[PDF_00837] English D., Cui Y., Siddiqui R. A. (1996). Messenger functions of phosphatidic acid.. Chem Phys Lipids.

[PDF_00839] Exton J. H. (1997). Cell signalling through guanine-nucleotide-binding regulatory proteins (G proteins) and phospholipases.. Eur J Biochem.

[PDF_00841] Khan P., Abbas S., Cheeseman S., Ranson M., McGown A. T. (1999). Development and validation of a sensitive solid-phase extraction and high-performance liquid chromatography assay for the novel antitumour agent CT2584 in human plasma.. J Chromatogr B Biomed Sci Appl.

[PDF_00845] Martin A., Gomez-Muñoz A., Waggoner D. W., Stone J. C., Brindley D. N. (1993). Decreased activities of phosphatidate phosphohydrolase and phospholipase D in ras and tyrosine kinase (fps) transformed fibroblasts.. J Biol Chem.

[PDF_00849] Siddiqui R. A., English D. (1997). Phosphatidic acid elicits calcium mobilization and actin polymerization through a tyrosine kinase-dependent process in human neutrophils: a mechanism for induction of chemotaxis.. Biochim Biophys Acta.

[PDF_00853] Siddiqui R. A., Yang Y. C. (1995). Interleukin-11 induces phosphatidic acid formation and activates MAP kinase in mouse 3T3-L1 cells.. Cell Signal.

[PDF_00862] Song J. G., Pfeffer L. M., Foster D. A. (1991). v-Src increases diacylglycerol levels via a type D phospholipase-mediated hydrolysis of phosphatidylcholine.. Mol Cell Biol.

[PDF_00859] Song J., Foster D. A. (1993). v-Src activates a unique phospholipase D activity that can be distinguished from the phospholipase D activity activated by phorbol esters.. Biochem J.

[PDF_00865] Waite K. A., Wallin R., Qualliotine-Mann D., McPhail L. C. (1997). Phosphatidic acid-mediated phosphorylation of the NADPH oxidase component p47-phox. Evidence that phosphatidic acid may activate a novel protein kinase.. J Biol Chem.

